# Molecular Genetics, Clinical Characteristics, and Treatment Outcomes of K_ATP_-Channel Neonatal Diabetes Mellitus in Vietnam National Children’s Hospital

**DOI:** 10.3389/fendo.2021.727083

**Published:** 2021-09-09

**Authors:** Can Thi Bich Ngoc, Tran Minh Dien, Elisa De Franco, Sian Ellard, Jayne A. L. Houghton, Nguyen Ngoc Lan, Bui Phuong Thao, Nguyen Ngoc Khanh, Sarah E. Flanagan, Maria E. Craig, Vu Chi Dung

**Affiliations:** ^1^Department of Endocrinology, Metabolism and Genetics, Vietnam National Children’s Hospital, Hanoi, Vietnam; ^2^Institute of Biomedical and Clinical Science, College of Medicine and Health, University of Exeter, Exeter, United Kingdom; ^3^Exeter Genomics Laboratory, Royal Devon & Exeter NHS Foundation Trust, Exeter, United Kingdom; ^4^Institute of Genome Research, Vietnam Academy of Science and Technology (VAST), Hanoi, Vietnam; ^5^Institute of Endocrinology and Diabetes, The Children’s Hospital at Westmead/Discipline of Child and Adolescent Health, University of Sydney, Sydney, NSW, Australia; ^6^School of Women’s and Children’s Health, University of New South Wales Medicine, Sydney, NSW, Australia

**Keywords:** neonatal diabetes mellitus, *ABCC8* mutations, *KCNJ11* mutations, sulfonylureas treatment in neonatal diabetes mellitus, diabetes mellitus in infants

## Abstract

**Background:**

Neonatal diabetes mellitus (NDM) is defined as insulin-requiring persistent hyperglycemia occurring within the first 6 months of life, which can result from mutations in at least 25 different genes. Activating heterozygous mutations in genes encoding either of the subunits of the ATP-sensitive K^+^ channel (K_ATP_ channel; *KCNJ11* or *ABCC8*) of the pancreatic beta cell are the most common cause of permanent NDM and the second most common cause of transient NDM. Patients with NDM caused by K_ATP_ channel mutations are sensitive to sulfonylurea (SU) treatment; therefore, their clinical management can be improved by replacing insulin with oral agents.

**Patients and Methods:**

Seventy patients were diagnosed with NDM between May 2008 and May 2021 at Vietnam National Children’s Hospital, and molecular genetic testing for all genes known to cause NDM was performed at the Exeter Genomic Laboratory, UK. Patients with *ABCC8* or *KCNJ11* mutations were transferred from insulin to oral SU. Clinical characteristics, molecular genetics, and annual data relating to glycemic control, SU dose, severe hypoglycemia, and side effects were collected. The main outcomes of interest were SU dose, SU failure (defined as permanent reintroduction of daily insulin), and glycemic control (HbA1c).

**Results:**

Fifty-four of 70 patients (77%) with NDM harbored a genetic mutation and of these; 27 (50%) had activating heterozygous mutations in *ABCC8* or *KCNJ11*. A total of 21 pathogenic mutations were identified in the 27 patients, including 13 mutations in *ABCC8* and 8 mutations in *KCNJ11*. Overall, 51% had low birth weight (below 3rd percentile), 23 (85%) were diagnosed before 3 months of age, and 23 (85%) presented with diabetic ketoacidosis. At diagnosis, clinical and biochemical findings (mean ± SD) were pH 7.16 ± 0.16; $\text{HC}{\text{O}}_{3}^{-}$, 7.9 ± 7.4 mmol/L; BE, −17.9 ± 9.1 mmol/L; HbA1C, 7.98% ± 2.93%; blood glucose, 36.2 ± 12.3 mmol/L; and C-peptide median, 0.09 (range, 0–1.61 nmol/l). Twenty-six patients were successfully transferred from insulin to SU therapy. In the remaining case, remission of diabetes occurred prior to transfer. Glycemic control on SU treatment was better than on insulin treatment: HbA1c and blood glucose level decreased from 7.58% ± 4.63% and 19.04 ± 14.09 mmol/L when treated with insulin to 5.8 ± 0.94% and 6.87 ± 3.46 mmol/L when treated with SU, respectively.

**Conclusions:**

This is the first case series of NDM patients with *ABCC8/KCNJ11* mutations reported in Vietnam. SU is safe in the short term for these patients and more effective than insulin therapy, consistent with all studies to date. This is relevant for populations where access to and cost of insulin are problematic, reinforcing the importance of genetic testing for NDM.

## Introduction

Neonatal diabetes mellitus (NDM) is defined as uncontrolled hyperglycemia with onset in the first 6 months of life. It is estimated to affect one in 90,000 newborns (1). NDM can be divided into three forms based on phenotypic characteristics; transient NDM (TNDM), permanent NDM (PNDM), and syndromic NDM (2). TNDM and PNDM account for 90% of NDM cases. NDM can result from mutations in at least 25 different genes (2). Most cases of TNDM are caused by imprinting defects on chromosome 6q24, with presentation in infancy, remission, and subsequent relapse in later life. Activating heterozygous mutations in the genes encoding either of the subunits of the ATP-sensitive K^+^ channel (K_ATP_ channel; *KCNJ11* or *ABCC8*) of the pancreatic beta cell are the second most common cause of TNDM (26% of TNDM cases) and the most common causes of PNDM (44% of PNDM cases) (2). The *KCNJ11* (MIM # 600937) and *ABCC8* (MIM # 60059) genes are located on the short arm of chromosome 11 (11p15.1) and encode the Kir6.2 subunit and the SU receptor 1 (SUR1) regulatory subunit of the K_ATP_ channel, respectively. In the normal pancreatic beta-cell, increased glucose enters the cell *via* a glucose transporter and is metabolized by the enzyme glucokinase, resulting in increased production of ATP. This causes closure of the K_ATP_ channel, which, in turn, depolarizes the cell membrane, activating the influx of calcium through voltage-gated calcium channels that subsequently allows exocytosis of insulin granules. Activating *KCNJ11* and *ABCC8* mutations cause the K_ATP_ channels to remain inappropriately open even in the presence of hyperglycemia. Without channel closure, the cell membrane is not able to depolarize effectively; thus, insulin cannot be released from the beta-cell (3).

In the clinical setting, insulin is the immediate choice for establishing glycemic control in NDM patients because it will be effective in all cases where an insulin deficit is involved. If a diagnosis of diabetes is made before 6 months of age and genetic screening is undertaken, the identification of mutations in *KCNJ11* or *ABCC8* provides an alternative therapeutic strategy. This is because if NDM results from overactive K_ATP_ channels, closing these channels is a key step to suppress insulin release, and sulfonylureas (SU) are well-studied K_ATP_ channel inhibitors (4). Moreover, SU have proven to be an effective treatment for individuals with NDM resulting from *KCNJ11* or *ABCC8* mutations (5, 6).

In 2020, De Franco et al. (7) collected and summarized a total of 748 *ABCC8* and 205 *KCNJ11* pathogenic and likely pathogenic mutations associated with congenital hyperinsulinism and NDM from various countries. In the present study, we report *KCNJ11/ABCC8* pathogenic mutations in Vietnamese patients with NDM diagnosed at Vietnam National Children’s Hospital between May 2008 and May 2021, and the outcomes of SU therapy transfer.

## Research Design and Methods

### Study Design and Individuals

The patients were diagnosed with NDM and were admitted to Vietnam National Children’s Hospital from May 2008 to May 2021. Inclusion criteria were hyperglycemia onset before 6 months of age and fasting blood glucose ≥126 mg/dl (7.0 mmol/L). Fasting was defined as no caloric intake for at least 4 h in children aged 0–1 years or random plasma glucose concentration ≥11.1 mmol/L (200 mg/dl). Hyperglycemia lasting at least 2 weeks required insulin for treatment. All patients with *KCNJ11* or *ABCC8* mutations and their parents agreed to participate in the study. Exclusion criteria were hyperglycemia due to glucose infusion, infection, stress, drugs, and other factors.

### Data Collection and Biochemical Analyses

Clinical phenotype and biochemical tests were performed at Vietnam National Children’s Hospital. Data included pedigree, sex, date of birth, gestational age, birth weight, date of diabetes diagnosis, natural history, and examination at diagnosis such as weight, height, and symptoms of diabetic ketoacidosis (DKA) including tachypnea, dehydration, lethargy, coma, and other symptoms such as convulsion. Blood glucose and HbA1C were measured by the automated Beckman Coulter AU2700/AU680 system. The specimen was collected in the early morning at schedule visits. Hexokinase technology with ORS 6221 reagent of OLYMPUS and ORS6192 reagent were used for blood glucose and HbA1c testing, respectively. Insulin and C-peptide were measured using immunoassay chemiluminiscent technology by automated biochemistry Hitachi 704. Arterial blood gas was measured by spectrometry, using GEM primer 300. Capillary glucose levels were measured at home by One Touch Ultrain in all patients five times/day (before breakfast, lunch, dinner, 22 h and 2 h) or whenever there was an abnormality. Continuous glucose monitoring (CGM) over 7 days was monitored by using the Medtronic Ipro™2 Professional. HbA1C was checked at 3-month intervals. Blood glucose level and HbA1C targets were determined according to the International Society of Pediatrics and Adolescent Diabetes (ISPAD) 2018 guideline (8).

### Mutation Analysis

Mutation analysis was performed at the Exeter genomic laboratory, UK. Blood samples were taken with informed consent obtained from the patients and their parents. Genomic DNA was extracted from peripheral blood using phenol/chloroform methods at Vietnam National Children’s Hospital.

The single exon of the *KCNJ11* gene was amplified in three overlapping fragments, as previously described (9). The *ABCC8* gene was analyzed at the same time as *KCNJ11*. The 39 exons of *ABCC8* were amplified in 38 fragments using previously described primers (10). PCR products were sequenced on an ABI 3100 or ABI 3730 (Applied Biosystems, Warrington, UK). Sequences were compared with the reference sequences (*KCNJ11*, NM_000525.3; *ABCC8*, NM_001287174.1) using Staden or Mutation Survey or software version 2.61.

The identified mutations were checked in common databases such as dbSNP154 database, ClinVar database, Leiden open variation database (LOVD), human gene mutation database (HGMD), and the genome aggregation database (gnomAD). *In silico* analysis was performed using Alamut Visual. Pathogenic variants identified after 2018 were classified using the ACMG best practice guidelines (11). Protein visualization was generated using the Protter website (http://wlab.ethz.ch/protter/start/)

### Transfer to Sulfonylureas From Insulin

For this study, clinicians were provided with two recommended protocols for the transfer to the SU glyburide (also known as glibenclamide) for Kir6.2 and SUR1 patients [see www.diabetesgenes.org and (12)]. One was for a rapid inpatient transfer, where the glyburide dose was increased by 0.2 mg/kg/day every day and the other for a slower outpatient transfer, where the glyburide dose was increased by 0.2 mg/kg/day every week. Both involved the gradual withdrawal of insulin, as the SU was introduced depending on blood glucose levels. These protocols were modified by the treating clinicians (5).

### Statistical Analysis

Data analyses were performed using SPSS version 12.0 (SPSS Inc., Chicago, IL). Data are expressed as frequency (%), mean ± SD, or median and range. Continuous variables were analyzed using ANOVA or Kruskal–Wallis tests. Proportions were compared using Fisher’s exact tests. Statistical significance was defined as *p* < 0.05 or the corresponding Bonferroni-adjusted *p* value for multiple comparisons.

## Results

A total of 70 patients were diagnosed with NDM before 6 months of age. Pathogenic variants were identified in 54 (77%). The most common genetic causes were mutations in *ABCC8* (26%) and *KCNJ11* (24%). The current study focused on the analysis of the 27 patients with *ABCC8/KCNJ11* mutations.

### Clinical Features of NDM Patients With *KCNJ11/ABCC8* Mutations

Of the 27 patients with a K_ATP_ channel mutation, 14 had a mutation in *ABCC8*, and 13 had a *KCNJ11* mutations. Clinical characteristics of these patients are provided in **Table 1**. Five of the 27 patients had TNDM, which was diagnosed before 3 months of age and onset with severe/moderate DKA. The average birth weight was 2758.3 ± 419.0 g with gestational age of 39.2 ± 1.28 weeks. Fifty-two percent (14/27) of the subjects were small for gestational age with a birth weight below the third centile. The average onset age in 27 cases was 60 ± 36.4 days, of which 85% were diagnosed before 3 months of age. At diagnosis, biochemical studies showed blood glucose, 36.2 ± 12.3 mmol/L; HbA1C, 7.16 ± 0.16% (normal range, 4–6.5%); $\text{HC}{\text{O}}_{3}^{-}$, 7.9 ± 7.4 mmol/L; BE, −17.9 ± 9.1 mmol/L; and C-peptide, median of 0.09 (range, 0–1.61 nmol/L; normal range, 0.26–0.62). Twenty-three patients (85.2%) presented with DKA. Nine patients (33.3%) had a convulsion at diagnosis.

**Table 1:** Clinical features of 27 NDM Vietnamese patients with *KCNJ11*/*ABCC8* mutations.

Pt	Age (days)	BW (percentile)	DKA	Glucose(mmol/L)	pH	$\text{HC}{\text{O}}_{3}^{-}$ (mmol/L)	BE (mmol/L)	HbA1C (%)	C-peptide(nmol/L)	Neurological symptoms	Type	Mutation
1	44	<3	Severe	49.5	7.12	–	−22.7	9.7	0.009	Convulsion	PNDM	*KCNJ11*: p.R201H
2	37	<3	Severe	31.2	6.9	1	Very low	8.4	0.05	DEND	PNDM	*KCNJ11*: p.R201C
3	45	<3	Mild	28.2	7.34	14.5	−9.5	5.8	0.3	No	PNDM	*ABCC8*: p.R1183W
4	36	<3	No	30.9	7.36	4	−18	8.0	0.2	DEND	PNDM	*ABCC8*: p.E747X
5	44	50	Severe	26.2	7.03	3.7	−25.1	10.3	0.03	No	PNDM	*ABCC8*: p.E128K/p.E747X
10	160	70	Severe	37.2	6.9	1.9	−28.2	13.7	–	No	TNDM	*KCNJ11*: p.R50Q
12	7	3	Moderate	20.6	7.2	20.6	−5.7	5.4	0.003	No	PNDM	*KCNJ11*: p.R201C
13	15	<3	Mild	22.4	7.3	12.8	−15.4	3.5	0.52	Mild mental development delay	PNDM	*ABCC8*: p.A1153G
14	96	<3	Severe	47.7	6.99	4.3	−26	6.7	0.04	No	PNDM	*ABCC8*: c.3403-1G>A/p.E1507Q
15	45	10	No	39.3	7.35	28.9	3.2	6.0	0.09	Convulsion	PNDM	*KCNJ11*: p.E292G
16	52	3	Severe	43.1	6,9	4	−28.7	5.1	0.0001	No	TNDM	*KCNJ11*: p.E229K
23	71	>10	Severe	25.6	7.1	6.3	−22.2	7.2	0.19	No	PNDM	*ABCC8*: p.C435R
24	36	>10	Severe	31.7	7,08	3.3	−26	7.6	0.17	No	TNDM	*ABCC8*: p.R1183W
25	48	3	No	13.08	7.44	23	−0.8	8.2	0.03	No	PNDM	*ABCC8*: p.P1199L
26	62	10	Severe	41.6	7.06	3.7	−26.6	10.2	0.1	No	PNDM	*KCNJ11*: p.R201H
27	82	10	Severe	30.0	6.89	5,1	Very low	11.5	0.01	No	TNDM	*ABCC8*: p.R1183W
28	72	3	Severe	27.7	6.86	3.6	-28.5	9.31	0.08	Convulsion	PNDM	*KCNJ11*: p.K185Q
30	100	<3	Moderate	56	7.2	5.4	−19.9	11.0	0.27	No	PNDM	*KCNJ11*: p.G53S
32	72	>10	severe	53.11	6.9	3	Very low	8.17	0.07	No	PNDM	*ABCC8*: p.R1380H
33	33	10	Mild	50.1	7.29	17.8	−8.8	4	0.09	No	PNDM	*ABCC8*: p.R598Q/p.R826W
35	62	50	No	27.8	7.38	10.1	−15	4.5	1.61	Convulsion	PNDM	*KCNJ11*: p.S331P
36	50	<3	Moderate	Very high	7.19	8.0	−19	7.58	0.41	Convulsion	TNDM	*ABCC8*: p.E1141G
37	81	>10	Severe	26.87	7.15	3.5	−25.8	12	0.36	Convulsion	PNDM	*KCNJ11*: p.R201H
40	30	<3	Mild	53.3	7.24	3.9	−21	5.1	0.27	Convulsion	PNDM	*ABCC8*: p.G833S
48	23	<3	Severe	59.56	7.05	2.6	−25.5	4.05	0.04	No	PNDM	*KCNJ11*: p.R201C
49	90	>10	Severe	27.8	7.04	3.0	NA	8.3	0.27	No	PNDM	*KCNJ11*: p.R201C
52	152	>10	Mild	40.2	7.31	NA	NA	14.3	0.33	No	PNDM	*ABCC8*: p.R1183W

(-), Not detected; NA, Not analysis.

### Molecular Genetic Analysis

**Figure 1:**
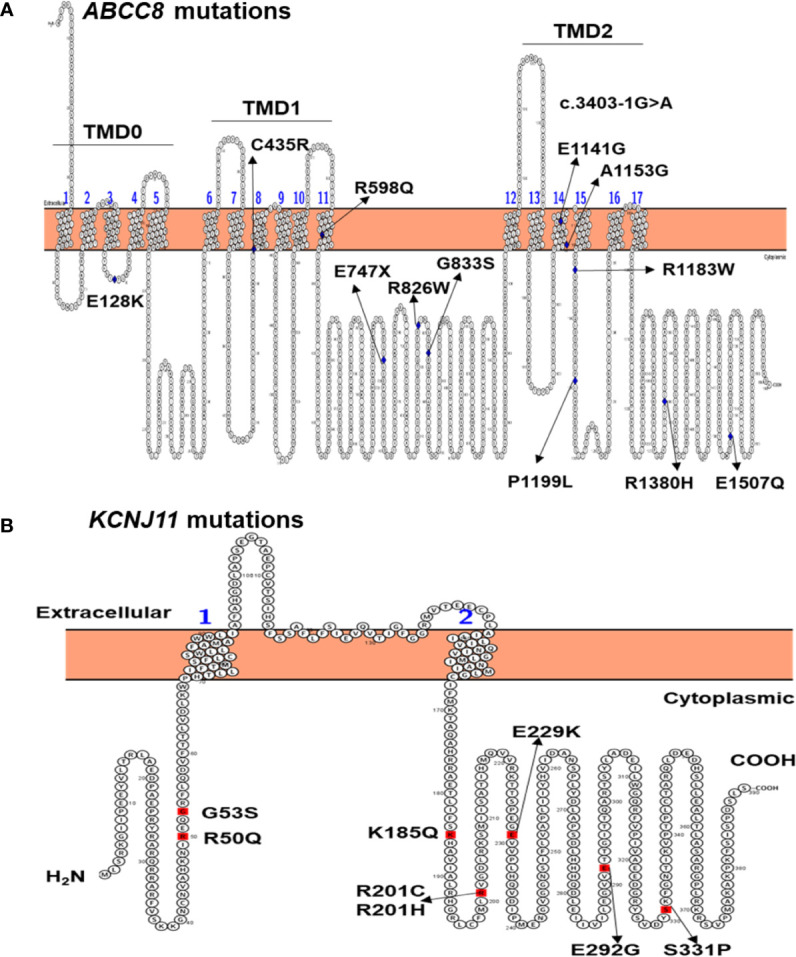
Location of *ABCC8* mutations in the SUR1 protein **(A)** and *KCNJ11* mutations in the Kir6.2 protein **(B)** identified in 27 Vietnamese patients with NDM. TMD, transmembrane domain.

A total of 21 pathogenic variants were identified, including 13 *ABCC8* and 8 *KCNJ11* mutations (**Table 2** and **Figure 1**). These mutations were predicted to be disease causing by the Mutation Taster tool and evaluated as pathogenic variants in the ClinVar database. Four mutations, namely, *ABCC8* (p.E747X), *ABCC8* (p.R1183W), *KCNJ11* (p.R201C), and *KCNJ11* (p.R201H), were found in multiple patients. Except for the *ABCC8* (p.E747X) mutation, which was homozygous in patient 4 (13), the remaining mutations were heterozygous.

**Table 2:** Molecular analyses of 27 Vietnamese patients with NDM.

Gene	cDNA change	Protein change	Zygosity	Inheritance	dbSNP	ClinVar	Reference	Type **(Patient No.)**	Treatment
*ABCC8*	c.382G>A	p.E128K	Het	Maternal	rs781617345	RCV001058712 Pathogenic	(13)	PNDM (5)	Ins→SU
*ABCC8*	c.1303T>C	p.C435R	Het	Paternal	–	Pathogenic	(14)	PNDM (23)	Ins→SU
*ABCC8*	c.1793G>A	p.R598Q	Het	Maternal	rs1344172059	VCV000523361.1 Likely pathogenic	unpublished	PNDM (33)	Ins→SU
*ABCC8*	c.2239G>T	p.E747X	Hom (4) Het (5)	Paternal and maternal (4) Paternal (5)	–	RCV001051901.2 Pathogenic	(13)	PNDM (4) PNDM (5)	Ins→SU
*ABCC8*	c.2476C>T	p.R826W	Het	*De novo*	–	Pathogenic	(15)	PNDM (33)	Ins→SU
*ABCC8*	c.2497G>A	p.G833S	Het	*De novo*	–	Pathogenic	(16)	PNDM (40)	Ins→SU
*ABCC8*	c.3403-1G>A	Splicing	Het	Maternal	rs576684889	VCV000370935.5 Likely Pathogenic	(17)	PNDM (14)	Ins→SU
*ABCC8*	c.3422A>G	p.E1141G	Het	Paternal	–	Pathogenic	unpublished	TNDM (36)	Ins→SU Remission 24 months
*ABCC8*	c.3458C>G	p.A1153G	Het	Maternal	–	Pathogenic	unpublished	PNDM (13)	Ins→SU
*ABCC8*	c.3547C>T	p.R1183W	Het	Paternal (3, 24) *De novo* (27, 52)	rs797045209	VCV000210076.2 Pathogenic	(18)	TNDM (24, 27) PNDM (3, 52)	Ins→SU Remission 6 months (24) Remission 14 months (27)
*ABCC8*	c.3596C>T	p.P1199L	Het	*De novo*	rs1554909277	VCV000434047.1 Pathogenic	(19)	PNDM (25)	Ins→SU
*ABCC8*	c.4139G>A	p.R1380H	Het	Maternal	rs193922401	VCV000585346 Likely Pathogenic	(15)	PNDM (32)	Ins→SU
*ABCC8*	c.4519G>C	p.E1507Q	Het	Paternal	–	Pathogenic	(20)	PNDM (14)	Ins→SU
*KCNJ11*	c.149G>A	p.R50Q	Het	*De novo*	rs80356611	VCV000036431.1 Pathogenic	(21)	TNDM (10)	Ins→SU Remission 6 months
*KCNJ11*	c.157G>A	p.G53S	Het	*De novo*	rs80356613	VCV000008681.1 Pathogenic	(22)	PNDM (30)	Ins→SU
*KCNJ11*	c.553A>C	p.K185Q	Het	*De novo*	–	Pathogenic	(23)	PNDM (28)	Ins→SU
*KCNJ11*	c.601C>T	p.R201C	Het	Maternal (2) De novo (12, 48, 49)	rs80356625	VCV000008668.3 Pathogenic	(24)	PNDM (2) PNDM (12, 48, 49)	Ins→SU
*KCNJ11*	c.602G>A	p.R201H	Het	*De novo*	rs80356624	VCV000008666.4 Pathogenic	(25)	PNDM (1, 26, 37)	Ins→SU
*KCNJ11*	c.685G>A	p.E229K	Het	*De novo*	rs587783673	RCV000146117 Pathogenic	–	TNDM (16)	Ins→SU Remission 50 months
*KCNJ11*	c.875A>G	p.E292G	Het	Maternal	–	Pathogenic	(26)	PNDM (15)	Ins→SU
*KCNJ11*	c.991T>C	p.S331P	Het	Paternal	–	Pathogenic Variant #0000497821 LOVD database	Unpublished	PNDM (35)	Ins→SU

Het, heterozygous; Hom, homozygous; PNDM, permanent neonatal diabetes mellitus; TNDM, transient neonatal diabetes mellitus; Ins, insulin; SU, sulfonylureas.

Twelve patients inherited mutations from their parents, while in 14 patients, the mutation had arisen *de novo*. Patient 33 inherited the *ABCC8* (p.R598Q) mutation from his mother and also had a *de novo* mutation *ABCC8* (p.R826W). Three patients 5, 14, and 33 had compound heterozygous mutations in *ABCC8*. Among 21 pathogenic mutations, four were unpublished, including *KCNJ11* (p.S331P), *ABCC8* (p.E1141G), and *ABCC8* (p.A1153G) (**Table 2**).

### Transfer to Sulfonylureas

**Figure 2:**
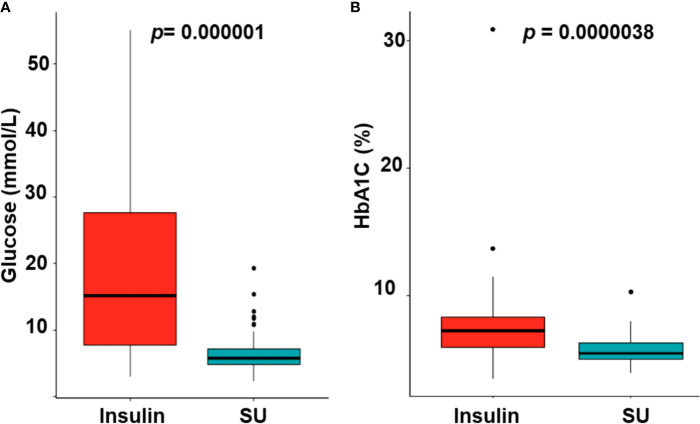
The glycemic control **(A)** and reduction of HbA1C levels **(B)** with switching from insulin to sulfonylurea therapy. SU, sulfonylurea.

Of the 27 patients with mutations in *KCNJ11*or *ABCC8*, 26 were successfully transferred from insulin to SU treatment. Patient 24 remitted after 5 months of insulin treatment and before transfer to SU. Glycemic fluctuations reduced when the patients were on SU treatment as compared with insulin treatment (**Figure 2A**, *p* = 0.000001). The mean HbA1C level dropped from 7.75% ± 3.60% on insulin treatment (13.3 ± 25 months) to 5.69% ± 1.02% on SU treatment (86.0 ± 56.9 months, p = 0.0000038) (**Figure 2B**). While on insulin treatment, there was one case of DKA and one case with convulsion due to severe hypoglycemia. After transfer to SU (86.0 ± 56.9 months), there were no patients with DKA or hypoglycemia.

Two of the 27 patients (patients 2 and 4) were diagnosed with developmental delay, epilepsy, and neonatal diabetes (DEND) syndrome (**Table 1**). Both individuals successfully transferred to SU, which resulted in improved mental and motor development. Patient 2, who is heterozygous for the *KCNJ11* p.R201C mutation, is currently 14 years of age and can understand simple sentences and has good eye contact. Patient 2 showed a decrease in HbA1c from 8.25% ± 0.20% (insulin treatment) to 6.65% ± 0.07% (SU treatment). During insulin treatment, patient 2 was admitted to the hospital twice (due to DKA and convulsion due to severe hypoglycemia). After transfer to SU, there were no further episodes of severe hypoglycemia or DKA. Patient 4 with a homozygous nonsense mutation in *ABCC8* p.(E747X) is currently 16 years of age and can walk, speak in short sentences, and understand and answer some simple questions. When patient 4 was treated with insulin, HbA1C was 8.5% ± 0.4%, and blood glucose ranged from 4 to 20 mmol/L; however, HbA1c decreased to 5.3% ± 0.2%, and blood glucose fluctuation ranged from 5 to 10 mmol/L when he was treated with SU.

Two TNDM patients with *KCNJ11* mutations (p.(R50Q) and p.(E229K)) and three TNDM patients with *ABCC8* mutations [two patients with p.(R1183W) and one patient with p.(E1141G)] remitted at 6, 50, 6, 14, and 24 months of ages, respectively (**Table 2**).

Treatment requirements (insulin or SU dose) did not show significant differences between the patients with *KCNJ11* mutations and those with *ABCC8* mutations (data not shown).

### Side Effects

None of the patients reported side effects during SU treatment such as diarrhea, nausea, or vomiting. Renal and liver function tests of all patients were checked every 6–12 months; all were within the normal range. No severe hypoglycemic episodes were reported while on SU treatment, and no other side effects were noted.

## Discussion

In our comprehensive mutation analysis of a large cohort of 70 patients with NDM enrolled at Vietnam National Children’s Hospital, we identified gene mutations in 54 cases (77%). In these 54 patients with a confirmed genetic diagnosis, mutations in the K_ATP_ channel genes was the most common cause of NDM with a rate of 50%. Overall, 51% had low birth weight (below third percentile), 23 (85%) were diagnosed before 3 months of age, and 23 (85%) presented with DKA. Twenty-six patients were successfully transferred from insulin to SU therapy, and glycemic control subsequently improved.

The mutation rate of 77% in our study was lower than that in Ukrainian (12) and Chinese studies but similar to the University of Chicago Monogenic Diabetes Registry (n = 88) (27). In contrast, Russo et al. (28) found the most common genes causing NDM diagnosed during the first 6 months of life were *KCNJ11* and *ABCC8* (70%), but mutations in *KCNJ11* were more common than *ABCC8*. These differences may be due to ethnicity, race, or size of the study cohort. In our study, we only investigated patients with NDM onset before 6 months of age who have mutations in the genes encoding the K_ATP_ channel.

The rate of patients with low birth weight (under third percentile) was 52%, which is similar to the results reported by Besser et al. (29). In the study reported by Russo et al. (28), the patients diagnosed with PNDM before 6 months of age but without mutations in *KCNJ11*, *ABCC8*, or *INS* had higher birth weight than those with mutations in these genes.

The majority of our cohort (85%) presented in DKA. Similarly, Letourneau et al. (27) reported that 66% of patients with neonatal diabetes (and 79% of patients with *KCNJ11/ABCC8* mutations) presented with DKA. While this is slightly less than observed in our cohort, there may have been a delay in diagnosis in some of our cases, which is reflected in the later age of diagnosis. This delay may be related to the challenge of diagnosing diabetes in infants who cannot communicate symptoms and in whom polydipsia and polyuria may not be readily apparent—indeed, this could even be reassuring to clinicians.

The numbers of Vietnamese patients with *ABCC8* and *KCNJ11* mutations were similar (14 versus 13), which is consistent with the findings in the Indian population (30). In contrast, Hashimoto et al. (31) reported more patients with *KCNJ11* than *ABCC8* mutations (16 versus 8). Recurrent mutations *KCNJ11* p.(R201C), *KCNJ11* p.(R201H), and *ABCC8* p.(R1183W) in Vietnamese patients have been reported in NDM patients in Jordan (32), India (30), Ukraine (12), The United States (33), Japan (34), and China (35). Therefore, these mutations may be considered as common mutations in different ethnic groups. The high rate of *de novo* mutations, which can arise either during gametogenesis or embryogenesis in NDM patients (15/27), identified in the current study, is consistent with the findings of Edghill et al. (36).

None of the 12 mutations identified in *ABCC8* are located in the glibenclamide binding pocket of SUR1. However, four mutations [*ABCC8* p.(C435R) and p.(R598Q), p.(E1141G), and p.(A1153G)] are located in the transmembrane domains TMD1 and TMD2, respectively (**Figure 1A**). Interestingly, p.(C435R) was previously reported in two TNDM patients (15, 37), while patient 23 in our study had PNDM after treatment on SU 72 months (**Table 1**). Three *ABCC8* mutations p.(R598Q), p.(E1141G), and p.(A1153G) are unpublished (**Table 2**). Patient 33 had a compound heterozygous *ABCC8* mutation, p.(R598Q) and p.(R826W) (**Tables 1**, ** 2**).

Eight *KCNJ11* mutations identified in this study are located in the N- and C-terminal regions (**Figure 1B**), which form the ATP binding pocket of Kir6.2 (38). The p.(R50Q), p.(K185Q), p.(R201C), and p.(R201H) mutations may reduce the response of the channel to ATP, as they lie at the main binding pocket (23, 24, 39). The *KCNJ11* p.R50Q mutation has been reported to cause both TNDM (34) and PNDM (9); therefore, patients with this mutation can present with different phenotypes as suggested by Suzuki et al. (34). The *KCNJ11* p.(K185Q) mutation was identified in PNDM patient 28 (**Table 1**). This mutation have been reported previously in a 3-year-old girl with PNDM, and after treatment with insulin, her HbA1C was between 6.8% and 7.8% (23). In our study, patient 28 was also treated with insulin; however, she was transferred to SU, resulting in an HbA1C of 7.6% and blood glucose level of 7.9 mmol/L. Functional studies indicated that this mutation reduced ATP binding to Kir6.2, resulting in a reduction in ATP sensitivity of the K_ATP_ channel, leading to PNDM in the patient (23).

SU therapy is effective in the treatment of hyperglycemia in patients with NDM who have a mutation in *KCNJ11* or *ABCC8*. Up to 90–95% of patients with NDM caused by mutations in these genes can cease insulin therapy after initiation of SU therapy (3, 5). In our study, the rate was higher, at 96.3%. The one remaining case (3.7%) had a remission of diabetes at 5 months of age before transfer to SU could be initiated. SU acts on the K_ATP_ channel to promote closure, allowing insulin to be released from the beta cells. Since SU therapy increases insulin release, there is a risk for hypoglycemia to occur. However, there was no severe hypoglycemia reported in our study. Excellent glycemic control was maintained after commencing SU therapy (**Figure 2A**), which is similar to other reports (6, 40). In the study of Bowman et al. (40), there were no reports of severe hypoglycemia in 809 patient-year follow up for the whole *KCNJ11* cohort, and 93% of the participants remained on SU therapy for the 10-year duration; however, a high rate (14%) of patients presented with mild and transient side effects of SU such as diarrhea, nausea, weight loss due to reduced appetite, and abdominal pain (40). Whereas, in our study, there was no side effects of SU, continued follow-up will be required to determine the long-term outcome of SU therapy in this group of patients. Interestingly, two patients (patients 2 and 4) with DEND syndrome were successfully transferred to SU treatment, which is consistent with previous studies (41, 42).

In conclusion, we found that all patients in our cohort with *ABCC8* and *KCNJ11* mutations could be successfully treated with oral SU treatment even if they had previously been treated with insulin. It is essential to perform rapid genetic testing for *ABCC8/KCNJ11* in any patient diagnosed with diabetes before 6 months of age, particularly given issues regarding access to and cost of insulin in some populations. On SU treatment, we observed that this therapy is safe in the short term for patients with K_ATP_ channel NDM.

## Data Availability Statement

The raw data supporting the conclusions of this article will be made available by the authors, without undue reservation.

## Ethics Statement

The studies involving human participants were reviewed and approved by Vietnam National Children’s Hospital IRB#1, 18/879 Lathanh, Dongda, Hanoi, Vietnam. Written informed consent to participate in this study was provided by the participants’ legal guardian/next of kin.

## Author Contributions

CN, TD, SE, and VD conceptualized, designed the study, and wrote and reviewed the manuscript. VD, CN, BT, and NNK provided patients’ clinical information, and MC reviewed/edited the manuscript. EF, SF, JH, and NNL analyzed data and wrote and reviewed the manuscript. All authors contributed to the article and approved the submitted version.

## Funding

This project was supported by grants from the Wellcome Trust and the Royal Society (Grant Number: 105636/Z/14/Z). EDF is a Diabetes UK RD Lawrence fellow. SF has a Sir Henry Dale Fellowship jointly funded by the Wellcome Trust and the Royal Society (105636/Z/14/Z). MC supported by a NHMEC practitioner fellowship (#1136735).

## Conflict of Interest

The authors declare that the research was conducted in the absence of any commercial or financial relationships that could be construed as a potential conflict of interest.

## Publisher’s Note

All claims expressed in this article are solely those of the authors and do not necessarily represent those of their affiliated organizations, or those of the publisher, the editors and the reviewers. Any product that may be evaluated in this article, or claim that may be made by its manufacturer, is not guaranteed or endorsed by the publisher.
